# Case Report: Bi-allelic missense variant in the desmocollin 3 gene causes hypotrichosis and recurrent skin vesicles

**DOI:** 10.3389/fgene.2022.994509

**Published:** 2022-08-17

**Authors:** Khalid Al Hawsawi, Mazin Al Jabri, Mazen S. Dajam, Bashaer Almahdi, Waseem K. Alhawsawi, Safdar Abbas, Abeer Al Tuwaijri, Muhammad Umair, Majid Alfadhel, Sultan Al-Khenaizan

**Affiliations:** ^1^ Dermatology Department, King Abdulaziz Hospital, Makkah, Saudi Arabia; ^2^ Dermatology Department, Hera General Hospital, Makkah, Saudi Arabia; ^3^ Dermatology Department, King Fahad Armed Forces Hospital, Jeddah, Saudi Arabia; ^4^ College of Medicine, King Saud Bin Abdulaziz University for Health Sciences (KSAU-HS), Ministry of National Guard Health Affairs (MNG-HA), Riyadh, Saudi Arabia; ^5^ Dermatology Department, King Fahad Hospital of The University, Al Khobar, Saudi Arabia; ^6^ Department of Biological Science, Dartmouth College, Hanover, NH, United States; ^7^ Medical Genomics Research Department, King Abdullah International Medical Research Center (KAIMRC), Ministry of National Guard Health Affairs (MNGH), King Saud Bin Abdulaziz University for Health Sciences, Ministry of National Guard Health Affairs (MNGH), Riyadh, Saudi Arabia; ^8^ Genetics and Precision Medicine Department, King Abdullah Specialized Children Hospital (KASCH), King Abdulaziz Medical City, Ministry of National Guard Health Affairs (MNG-HA), Riyadh, Saudi Arabia; ^9^ College of Medicine, King Saud Bin Abdulaziz University for Health Sciences (KSAU-HS), Riyadh, Saudi Arabia; ^10^ Department of Dermatology, King Abdulaziz Medical City, Ministry of National Guard Health Affairs (MNG-HA), Riyadh, Saudi Arabia

**Keywords:** DSC3, HYPTSV, missense variant, novel mutation, saudi patient

## Abstract

**Background:** Hypotrichosis with Recurrent Skin Vesicles (HYPTSV) is an extremely rare condition, having autosomal recessive inheritance. Here in we report a 4-years- old Saudi boy who presented with a history of recurrent skin blisters that are localized to the extremities and hypotrichosis since birth.

**Methods:** The present study describes a consanguineous Saudi family segregating HYPTSV in an autosomal recessive fashion. A single proband (II-1) exhibited features such as diffused non-scarring alopecia on the scalp, intraepidermal blister, post-inflammatory hyperpigmented macules, and follicular hyperkeratosis. DNA of the index was subjected to whole-genome sequencing (WGS). Furthermore, 3D protein modeling was performed for the mutated and normal protein.

**Results:** WGS revealed a novel bi-allelic missense variant (c.154G>C; p. Val52Leu) in the *DSC3* gene, which segregated perfectly using Sanger sequencing. In addition, 3D protein modeling revealed a substantial change in the mutated DSC3 protein as compared to the normal DSC3 protein.

**Conclusion:** This is the 3rd novel variant reported in the *DSC3* gene associated with the HYPTSV phenotype. This report further strengthens the evidence that bi-allelic variants in the *DSC3* cause severe HYPTSV in humans.

## Introduction

Desmosomes are specialized adhesive protein complexes that localize to intercellular junctions whose primary function is cell adhesion and maintaining the integrity of the tissues (Najor, 2018). Specific tissues are rich in desmosomes specialized in mechanical stress, including the bladder, myocardium, *epidermis*, gastrointestinal mucosa, and epithelial integrity (Petrof et al., 2012; Samuelov et al., 2015).

Desmosomes are composed of intra-cytoplasmic plaque and transmembrane-spanning cadherins. The transmembrane machinery includes three types of desmocollins (DSC1, DSC2, and DSC3) and four desmogleins (DSG1, DSG2, DSG3, and DSG4). The intra-cytoplasmic plaque facilitates linking cadherins with intermediate filaments with the help of plakophilins, plakoglobin, and desmoplakin (Nekrasova1 and Green, 2013).

Localized in desmosomes are the desmocollins (DSC), classified as type-1 transmembrane glycoproteins formed in epithelial cells and involved in cell adhesion junctions. As a result of alternative splicing, three DSCs (DSC1–3) are formed, resulting in the generation of the DSC “a” and “b” isoforms. These two isoforms differ in the length of their respective carboxy-terminal domains (Petrof et al., 2012). Desmocollin 3 (DSC3) is a transmembrane core of desmosomes involved in heterophilic and homophilic adhesive interactions (Samuelov et al., 2015).

Bi-allelic mutations in the *DSC3* gene (OMIM 600271) have been reported to cause hypotrichosis and recurrent skin vesicles (HYPTSV) in humans. HYPTSV is a rare disorder characterized by sparse to absent scalp hair, eyelashes, eyebrows, body hair, and recurrent scalp and skin vesicles (OMIM 613102). HYPTSV is inherited in an autosomal recessive fashion. However, only two families exhibiting HYPTSV have been reported in literature having Bi-allelic variants in the *DSC3* gene (Ayub et al., 2009; Onoufriadis et al., 2020).

In the present study, we clinically and genetically characterize a consanguineous Saudi family displaying the hallmarks of HYPTSV. Furthermore, molecular analysis revealed a novel homozygous missense *DSC3* variant.

## Materials and methods

### Study approval and DNA extraction

The present study was approved by the IRB of KAIMRC, Riyadh, Saudi Arabia. The patients underwent a full clinical assessment for genodermatosis in the respective specialized hospital. In addition, standard written informed consent was obtained to publish clinical data and photographs from the affected individual’s parents.

Peripheral blood samples were collected from all the six family members, including the index (II-1), three healthy sisters (II-2, II-3, and II-4), and both parents I-1, I-2). The genomic DNA was extracted and quantified using standard methods (Massadeh et al., 2019).

### Whole genome sequencing

WGS was commercially performed at the renowned Centogene lab (Rostock, Germany). Briefly, genomic DNA was enzymatically fragmented, and libraries were generated by PCR-mediated addition of Illumina compatible adapters that were paired-end sequenced on an Illumina HiSeqX platform (Illumina, SanDiego, CA, United States) (average yield coverage depth of ∼30x) using Burrows-Wheeler Aligner (http://bio-bwa.sourceforge.net), and all reads were aligned against human assembly hg19 (GRCh37/hg19). Enrichment was carried out using SureSelectXT Human kit version 5 (Agilent Technologies, Santa Clara,CA, United States). SAMtools (http://samtools.sourceforge.net/) and PINDEL (http://gmt.genome.wustl.edu/packages/pindel/) were used for variant calling. Subsequently, filtering of the variants was performed with the help of the SAM tools varFilter and custom scripts. A standard bioinformatics pipeline was applied, including base pair calling, filtering out low-quality reads and possible artifacts, and variants annotation.

Structural variant (SV) calling is based on the DRAGEN pipeline from Illumina. All the variants obtained after filtering were then analyzed using Illumina Base space tool. Since the pedigree clearly depicts autosomal recessive inheritance, therefore, we queried the database to show bi-allelic or compound heterozygous for initial screening. However, all moods of inheritances were used for analysis. All disease-causing variants reported in the HGMD and ClinVar were considered. Furthermore, all variants in the gnomAD database with a minor allele frequency of less than 1% were considered (Alhamoudi et al., 2020; Asiri et al., 2021).

Filtration steps focused on coding exons and their flanking intronic regions, considering all the possible inheritance patterns. Furthermore, pedigree exhibiting the inheritance pattern, clinical information of the patient, and brief family history obtained from the parents and previous medical records were used for identified variants prioritization. Finally, the filtered variants were classified into five classes given by ACMG guidelines (Pathogenic; Likely pathogenic; VUS; Likely benign; Benign).

### Pathogenicity analysis

The potential effect of the identified variant was predicted using MutationTaster, Varsome, SIFT, Polyphen2, Mutation assessor, and FATHMM. The conservation of the amino acid was checked using NCBI [Homologene]. To check the frequency of the variant in general population, GnomAD, Exome Variant Server, 1,000 Genomes, ExAC, dbSNP and in-house database (https://kgd.kaimrc.med.sa/login/) were used.

### Mutation validation and sanger sequencing analysis

Using standard methods, bi-directional Sanger sequencing was used to confirm variant segregation in all the available family members (Alfadhel et al., 2021). The primers used for Sanger sequencing were designed using Primer3 software.

### Protein 3D modeling

The partial DSC 3 amino acid (890 aa which includes Cadherin 1-5 domain) sequence was retrieved from the UniProt Knowledgebase database (UniProtKB Accession: Q14574-1). Comparative protein modeling is one of the most precise computational approaches to predicting a consistent 3D structure from available information (Umair et al., 2019). The 3D structure of Human DSC3 protein was predicted by I-Tasser (https://zhanggroup.org/I-TASSER/). The structure of the mutated DSC 3 protein was generated using the MODELLER (9.19) tool. The MODELLER assists in the 3D structure prediction of proteins by satisfying spatial restraints, and the generated protein model was selected based on the MODELLER evaluation score. ChimeraX 1.4 was used for interactive visualization and analysis of molecular structures (Figures 2A–E).

### Model evaluation

A major problem in structural bioinformatics is the recognition of experimental errors and theoretical errors in the protein structural models. The obtained protein structure was assessed using different evaluation tools and was processed by RAMPAGE and ERRAT. Ramachandran plot generated using the RAMPAGE assists the models along with distribution of residues, while the ERRAT plot indicates the confidence and overall quality of the predicted model.

## Results

### Case presentation

**FIGURE 1:**
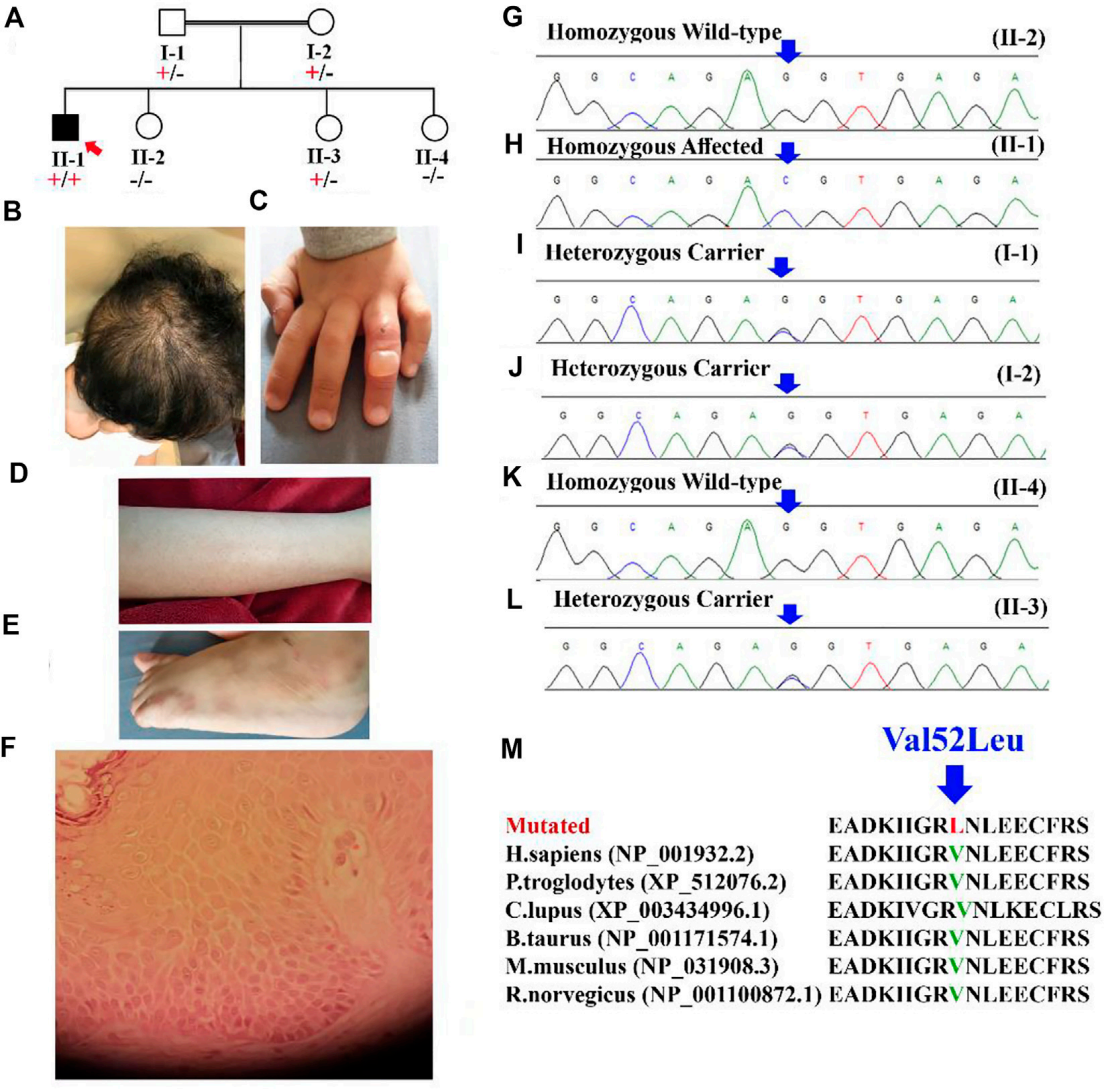
**(A)** Pedigree of the family showing autosomal recessive inheritance. The red arrow represents the index (II-1). **(B)** Scalp of the patient (II-1) showing unruly hair with diffuse non-scarring alopecia. **(C)** The dorsum of the fourth finger of the patient shows an intact blister. **(D)** Multiple tiny follicular papules were observed on the patient’s leg. **(E)** Postinflammatory hyperpigmented macules on the dorsum of the patient’s left foot (sites of previous blisters). **(F)** Skin biopsy from edge of a bulla on dorsum of foot showing the *epidermis* with increase in the spaces between keratinocytes. **(G–L)** Sanger sequencing electrograms showing bi-allelic wild, bi-allelic affected and heterozygous carrier. **(M)** Showing conservation of [Val52] amino acid across several species.

A 4-year-old Saudi boy was presented to the clinic with a history of recurrent skin blisters that were localized to the extremities since birth. The parents are consanguineous (first-degree cousins), and no family history of such disorder has been reported (Figure 1A). He was born with normal hair until the age of 1 year, when he began to have progressive hair loss until the age of 2 years, after which the hair fall improved but remained but was less severe than before the age of 2 years.

Eyelashes and brows were absent until he was 3 years old, when he began to grow a sparse eyelashes and brows but still experienced hair loss. (Figure 1B); otherwise, the boy was healthy. A review of systems, prenatal, natal and postnatal history was unremarkable. Skin examination revealed a single bulla over the dorsum of the fourth finger of the left hand (Figure 1C) and post inflammatory hyperpigmented macules on the dorsum of the patient’s left foot (sites of previous blisters) (Figures 1D,E). There were multiple tiny follicular papules scattered on all extremities. Scalp examination revealed unruly hair with diffuse nonscarring alopecia (Figure 1B). Eyelashes and eyebrows were sparse. The mucus membranes, nails, palms, and soles examinations were also unremarkable. An examination of the teeth was normal. The differential diagnosis includes hypotrichosis with recurrent Skin vesicles syndrome (H-RSVS), ectodermal dysplasia-skin fragility syndrome (ED-SFS), and skin fragility/woolly hair syndrome (SF-WHS). Trichoscopic examination of the hair was normal. A skin biopsy was taken from the blister, which showed an intraepidermal blister (Figure 1F). Detail comparative clinical description of patients in the present study with those reported in the literature is presented in Table 1.

**TABLE 1:** Comparative clinical description of patients reported to-date.

Clinical phenotypes	(Ayub et al., 2009)	(Onoufriadis et al., 2020)	Hawsawi et al. (Present study)
Sex	3 Females: 1 Male	Male	Male
Origin	Pakistan	Egyptian	Saudi Arabia
Consanguinity	+	+	+
Pregnancy event	Normal	Normal	Normal
Scalp hypotrichosis	+	+	+
Sparse eyebrows	+	+	+
Follicular papules	+	+	+
Inflammatory blisters	+	+	+
Sparse scalp hair	+	+	+
Skin vesicles with thin watery fluid	−	+	+
Trauma-induced bullae and crusted erosions	−	+	−
Follicular hyperkeratosis on the scalp, trunk, and extremities	−	+	+
Cracked lips with angular cheilitis	−	+	−
Leukonychia- nails	−	+	−
Dental anomalies	−	+	−
Inverted nipple	−	+	−
Mild- syndactyly	−	+	−
Clinodactyly	−	+	−
Age at last exam	N.A	5 years	3 years
Skeletal survey	−	−	−
Hearing test	−	−	−
Eye exam	−	−	−
Echocardiogram	Normal	Normal	Normal
Muscular issues	−	−	−
Chromosomal analysis	Normal	Normal	Normal
Zygosity	Bi-allelic	Bi-allelic	Bi-allelic
Type of mutation	Nonsense	Nonsense	Missense
Genetic results	*DSC3*: c.2129T>G; p.Leu710*	*DSC3*: c.2180T>G; p.Leu727*	*DSC3:* c.154G>C; p.Val52Leu

### Molecular analysis

After performing WGS using standard methods, variant filtration was performed. Variant filtration was based on the family pedigree-inheritance pattern (Figure 1A) and the patient’s clinical presentation (Figures 1B,E). As pedigree depicted autosomal recessive inheritance pattern, bi-allelic and compound heterozygous variants that were rare and disease-causing were screened (Alfadhel et al., 2019; Alhamoudi et al., 2021). Initial screening was performed using OMIM, HGMD, and variants were further classified according to ACMG guidelines.

After careful filtration we identified a bi-allelic missense variant (c.154G>C; p.Val52Leu), in the exon two of the *DSC3* gene (NM_001941.4; Chr 18q12.1). The identified variant (c.154G>C) was screened in all the family members using Sanger sequencing (Figure 1G–L). According to OMIM, only the *DSC3* variant identified in the present study could explain the disease phenotype in the patients investigated in the present study. The variant (c.154G>C) was not reported in the bi-allelic state in 1000genomes, gnomAD, and ExAC databases, and in-house WES/WGS database. The identified variant was also screened in 2000 control samples and was not identified outside the family, respectively (https://kgd.kaimrc.med.sa/welcome/). The variant was not identified in any of the samples outside the family even in heterozygous state. Furthermore, the Val52 amino acid was conserved across several species (Figure 1M).

### Pathogenicity index of c.154G>C p.Val52Leu

Using different tools (Table 2), the pathogenicity index of identified variant c.154G>C; p.Val52Leu was calculated, and the variant was classified as a variant of unknown significance (VUS; Class 3) according to the ACMG guidelines.

**TABLE 2:** Pathogenicity of the identified *DSC3* variant (c.154G>C; p.Val52Leu).

Tool used	Pathogenicity prediction	Score
*SIFT*	Damaging	0
*PROVEAN*	Damaging	−2.46
*MutationTaster*	Disease causing	0.9999
*MutPred*	Pathogenic	0.746
*FATHMM*	Damaging	0.7668
*EIGEN*	Pathogenic	0.8312
*MetaRNN*	Damaging	0.8289
*Mutation assessor*	Medium	3.005
*Varsome*	PM2, PP3, and VUS	PM2, PP3

### Protein 3D modeling

**FIGURE 2:**
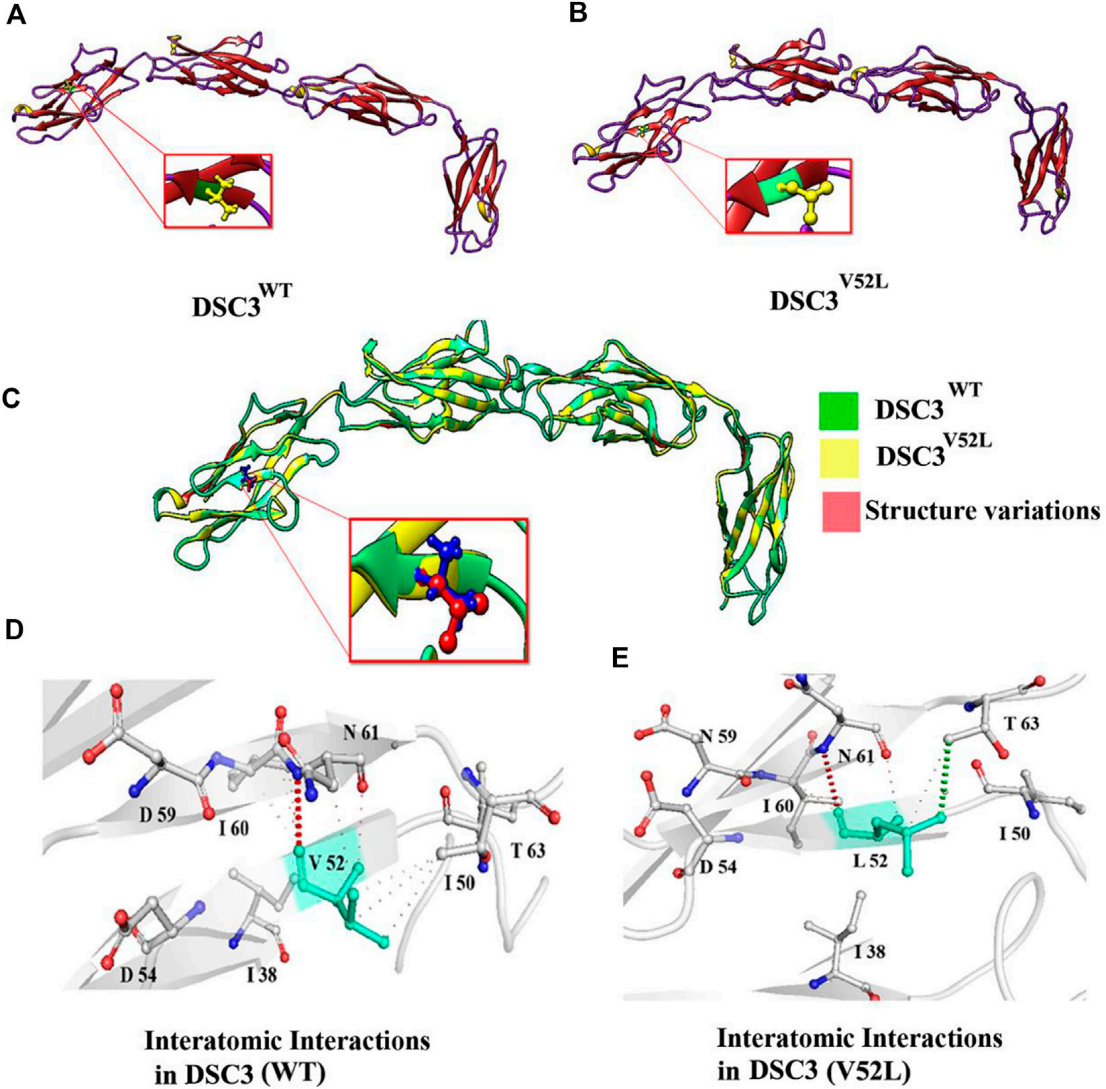
Three-dimensional structure of Desmocollin-3 protein and Interatomic interaction of Val52 and Leu52 with surrounding residues in both wild type and mutant Desmocollin3. **(A,B)** Representing structural 3D modeling for the DSC3^WT^ and DSC3^MU^ proteins. **(C)** Illustrating the superposed structure of DSC3^WT^ (green) and DSC3^MU^ (yellow) structures shows the difference (red) in overall confirmation due to the p. Val52Leu mutation. **(D)** Representing interatomic interaction of Val52 with surrounding residues in the DSC3^WT^ protein. **(E)** Showing the interatomic interaction of Leu52 with surrounding residues in DSC3^MU^ (p.Val52Leu) protein.

Using the MODELLER server, the 3D structure of DSC3 was modeled having a respectable degree of accuracy. The 3D models of wild type (p.Val52) and mutated DSC3 protein (p.Val52Leu) were predicted and evaluated. Ramachandran plot indicated 92%–93% of the wild type, and mutant structure residues lie in the acceptable regions of torsion angles (Figures 2A–C). In addition, the Errat provided an overall satisfactory quality factor of the wild type and mutant structure model (92% and 90%).

The substitution of Valine residue (p.Val52Leu) at this position might affect the secondary structure of the DSC3 protein. Structural analysis revealed that Val52 interacts with Ile50, Ile60, and Asp61. Although both Valine and Leucine contain a non-polar neutral side chain, the substitution of smaller Valine for bigger Leucine substantially disturbs surrounding amino acid interactions. The number of alkyl groups also influences the polarity. The more alkyl groups present, the more non-polar will be the amino acid. This effect makes leucine is more non-polar than valine. It results in potential new interactions that might disrupt the function and structure of the DSC3 protein. Using mCSM, ENCoM, and DUET, a −0.182, 0.116, and −0.714 kcal/mol change in the ΔΔG was observed for Val52Leu mutation (Figures 2D,E). These findings suggest that Val52Leu might disrupt DSC3 protein structure and function.

## Discussion

Herein, we report a single affected individual (II-1) exhibiting hallmark HYPTSV features such as scalp hypotrichosis, sparse scalp hair, sparse eyebrows, follicular papules, inflammatory blisters, and skin vesicles with thin watery fluid. The phenotypes observed in the present case overlap with those reported previously (Ayub et al., 2009; Onoufriadis et al., 2020). Features such as cracked lips with angular cheilitis, leukonychia-nails, dental anomalies, inverted nipple, mild-syndactyly and clinodactyly reported by Onoufriadis et al. (2020), were not observed in our patient (Table 1). The patient described by Onoufriadis et al. (2020) revealed widespread trauma-induced blisters similar to the index reported here, which is similar to the findings in the DSC3 knockout mouse model (Chen et al., 2008). A detailed clinical comparison is presented in Table 1.

Ayub et al. (2009), for the first time, described HYPTSV in a large family with four affected individuals exhibiting features of hereditary hypotrichosis of the scalp, eyelashes, eyebrows, axillary hair, skin vesicles, and follicular hyperkeratosis on the scalp. They revealed a Bi-allelic nonsense variant [c.2129T>G; p (Leu710*)] in the *DSC3* gene Ayub et al. (2009). In addition, a 5-year-old boy from Egypt, having consanguineous parents, was reported exhibiting a bi-allelic nonsense variant (c.2180T>G; p.Leu727*) in the *DSC3* gene. The affected individual revealed hypertrichosis and blisters on the feet, hands, and knees. In addition, the patient had sparse scalp hair and eyebrows, dry skin, cracked lips, angular cheilitis, hypotrichosis, blisters on extremities follicular hyperkeratosis (Onoufriadis et al., 2020).

Using WGS coupled with Sanger sequencing, we identified a bi-allelic missense variant (c.154G>C; p.Val52Leu) in the exon two of the *DSC3* gene. The variant results in the substitution of Valine amino acid at position 52 into Leucine. The identified variant is not observed in the bi-allelic state in several online databases such as 1000genomes, ExAC, and gnomAD. The Val52 amino acid is conserved across several species indicating its important role (Figure 1M). Protein 3D homology modeling revealed that the identified variant [p.Val52Leu], causes substantial changes in the overall DSC3 secondary structure and thus might cause functional damage.

As the previous reports suggested, loss of function variants causing the HYPTSV. However, here we associate a missense variant in the DSC3 gene that might cause HYPTSV in our patient. First, we have overlapping phenotypes with already reported patients in the literature. Secondly, change in single amino acid have been associated with severe disorders that might either change the charges, thus altering the intra- or intermolecular interactions, ligand binding, proper folding, affecting protein-protein interactions, disrupting electrostatic interactions, and effect charges on the side chains (Zhou and Pang, 2018). Thus, single amino acid substitution might change the interatomic interactions possibly leading to disruption of structure and/or function of the wild-type protein.

DSC3 is a constituent of the core transmembrane desmosomes and is associated with heterophilic and homophilic adhesive interactions in the intercellular space resulting in cell adhesion (Spindler et al., 2009). Furthermore, the DSC3 cytoplasmic domain interacts with the plakophilin(s) (PKP) and armadillo proteins plakoglobin (JUP), in particular plakophilin 3 (PKP3), which helps DSC3 to link to the intermediate filament cytoskeleton (keratin; KRT) *via* the adaptor protein desmoplakin (DSP) (Bonne et al., 2003).

DSC3 is crucial for mouse development as germline DSC3 null mutations are embryonically lethal (Den et al., 2006). Similarly, DSC3 null mutation in stratified epithelia revealed desmosomes that were unable to sustain cell adhesion (Chen et al., 2008). In addition, DSC3 mutant mice display skin erosions that might be due to the loss of coherence between epidermal cells due to the breakdown of intercellular bridges (Chen et al., 2008).

Parents having genetic skin conditions can be subjected to parenteral diagnosis, which can be accomplished by prenatal genetic testing for monogenetic disorders (PGT-M). PGT and *in vitro* fertilization are options for parents wishing to have future pregnancies (Alyafee et al., 2021a; Alyafee et al., 2021b). Although there is no specific management in these cases, patients are treated with supportive treatment. Skin blisters are treated by the concerned clinicians using calamine lotion, non-steroidal anti-inflammatory drugs (NSAIDs), such as Advil (ibuprofen), Aleve (naproxen), and aspirin.

In conclusion, we report the first case of HYPTSV from the Saudi population due to a bi-allelic variant in the DSC3 gene. This study provides additional evidence that variants in the DSC3 cause hypotrichosis and skin blisters in humans. Furthermore, the current report further expands the DSC3 mutation spectrum, which might help in genotype-phenotype correlations in the future.

## Data Availability

The datasets presented in this study can be found in online repositories. The names of the repository/repositories and accession number(s) can be found in the article/supplementary material.
